# The Real-World Endocrine Toxicity Profile of ICIs, VEGFR-TKIs, and Their Combination: Analysis of the FDA Adverse Event Reporting System (FAERS) Database

**DOI:** 10.32604/or.2026.074672

**Published:** 2026-04-22

**Authors:** Nicola Marrano, Mariangela Caporusso, Cosimo Matino, Irene Caruso, Carlo Ganini, Mimma Rizzo, Ludovico Di Gioia, Angelo Cignarelli, Sebastio Perrini, Luigi Laviola, Camillo Porta, Francesco Giorgino, Annalisa Natalicchio

**Affiliations:** 1 Section of Internal Medicine, Endocrinology, Andrology and Metabolic Diseases, Department of Precision and Regenerative Medicine and Ionian Area, University of Bari Aldo Moro, Bari, Italy; 2 Endocrinology Unit, Regional General Hospital “Francesco Miulli”, Acquaviva delle Fonti, Bari, Italy; 3 Endocrinology Unit, University Hospital “Consorziale Policlinico” of Bari, Bari, Italy; 4 Division of Medical Oncology, Interdisciplinary Department of Medicine, University of Bari Aldo Moro, Bari, Italy; 5 Division of Medical Oncology, University Hospital “Consorziale Policlinico” of Bari, Bari, Italy; 6 Section of Endocrinology, Department of Medicine and Surgery, LUM University, Casamassima, Bari, Italy

**Keywords:** Immune checkpoint inhibitor (ICI), vascular endothelial growth factor receptor tyrosine kinase inhibitor (VEGFR-TKI), endocrine immune-related adverse events (irAEs), food and drug administration adverse reporting system (FAERS), pharmacovigilance

## Abstract

**Background:**

Immune checkpoint inhibitors (ICIs) are a cornerstone of systemic therapy for renal cell carcinoma (RCC), used both in the adjuvant and metastatic settings across various lines of treatment, often in combination with vascular endothelial growth factor receptor tyrosine kinase inhibitors (VEGFR-TKIs). These therapies are associated with endocrine immune-related adverse events (irAEs), which can be irreversible and life-threatening if not promptly managed. Using data from the Food and Drug Administration Adverse Reporting System (FAERS), this study aimed to evaluate the real-world occurrence of endocrine irAEs in all approved VEGFR-TKI + ICI combinations for RCC, and to compare these findings with the corresponding VEGFR-TKI or ICI monotherapies. The immune doublet ipilimumab + nivolumab was not considered in this analysis.

**Methods:**

FAERS database from 2019 Q1 to 2024 Q2 was queried using OpenVigil 2.1-MedDRA-v24 and AERS*Mine* to identify endocrine irAEs reports. Reports were filtered by age, gender, and report severity. The frequency of reported endocrine irAEs associated with VEGFR-TKI + ICI combination therapies was compared to that reported for VEGFR-TKI or ICI monotherapy.

**Results:**

Compared with VEGFR-TKI monotherapies, VEGFR-TKI + ICI combinations showed a significant disproportionate reporting of endocrine irAEs, mostly associated with the combination regimens. In contrast, when compared with ICI monotherapy, VEGFR-TKI + ICI showed more heterogeneous disproportionality signals, with generally lower reporting of hypothalamus, pituitary, and hyperglycemic disorders, whereas hypoglycemia and thyroid irAEs were more frequently reported, except for autoimmune thyroid diseases.

**Conclusion:**

Combination therapy, compared with VEGFR-TKI monotherapy, was associated with a higher reporting frequency of specific endocrine irAEs, whereas comparisons with ICI monotherapy yielded mixed signals, highlighting regimen- and event-specific differences.

## 1 Introduction

Over the past decade, immune checkpoint inhibitors (ICIs)-including programmed death-1 (PD-1), programmed death ligand-1 (PD-L1), and cytotoxic T-lymphocyte antigen-4 (CTLA-4) inhibitors-have revolutionized the treatment of advanced malignancies. By blocking inhibitory signaling pathways that normally restrain T-cell activation and maintain immune tolerance, ICIs reinvigorate exhausted cytotoxic T cells and unleash durable anti-tumor immune responses [1]. Despite these advances, however, only 20%–30% of patients achieve meaningful and lasting clinical benefit [1–3]. Recent studies have highlighted the critical role of the tumor microenvironment (TME) and extracellular factors in shaping immune responses and modulating the efficacy of immunotherapies [4]. For instance, degradation of collagen has been proposed as a strategy to overcome immunosuppression and synergize with anti-PD-L1 therapy [4]. Additionally, the TME may be enriched with exosomal miRNAs that promote cancer aggressiveness and facilitate immune escape [5]. Abnormal tumor vasculature within the TME represents a major barrier to ICI efficacy, profoundly influencing anti-cancer immune activity. Pathologic angiogenesis limits immune-cell infiltration and promotes immune escape, while immunosuppressive cells in turn fuel further aberrant vessel growth, creating a vicious cycle that weakens anti-tumor immunity and drives cancer progression [1–3]. A central mediator of this process is vascular endothelial growth factor (VEGF). Beyond stimulating tumor vascularization, VEGF fosters profound immunosuppression by impairing dendritic cell maturation, promoting regulatory and myeloid-derived suppressor cells, and upregulating immune checkpoints [3,6,7]. Targeting this dysregulated angiogenesis with anti-angiogenic agents, mainly vascular endothelial growth factor receptor tyrosine kinase inhibitors (VEGFR-TKIs), has therefore emerged as a promising strategy to enhance immunotherapy. By normalizing tumor vasculature, VEGFR-TKIs improve immune-cell trafficking and counteract VEGF-driven immunosuppression, thereby potentiating the activity of ICIs [1–3].

Within this context, renal cell carcinoma (RCC) provides a particularly compelling model for VEGFR-TKI + ICI combination therapy. Renal cancer is the 14th most common malignancy worldwide [8], with over 465,000 new diagnoses reported in 2022–2025 [9], and RCC accounts for approximately 90% of these cases [8–10]. Alarmingly, nearly one-third of patients present with advanced or metastatic disease (mRCC) at diagnosis [11]. Among solid tumors, mRCC has demonstrated some of the most striking survival gains with the introduction of ICIs and VEGFR-TKIs, which, as a whole, have markedly prolonged both progression-free (PFS) and overall survival (OS) and redefined the therapeutic standard [12,13]. Importantly, most RCCs harbor *VHL* gene alterations, leading to hypoxia-inducible factor (HIF) activation and overproduction of VEGF and other pro-angiogenic cytokines [14,15]. This molecular profile makes RCC highly dependent on aberrant angiogenesis and an ideal candidate for VEGFR-TKI + ICI combinations. Reflecting this biology, ICIs and VEGFR-TKIs are currently used as monotherapy, or in combination in different treatment lines; for example, current first-line regimens for mRCC include the following combinations of a VEGFR-TKI and an ICI: axitinib plus pembrolizumab [AP], cabozantinib plus nivolumab [CN], or lenvatinib plus pembrolizumab [LP] [12,16,17]. Furthermore, the ICI pembrolizumab is presently used also in the adjuvant setting, to prevent disease recurrence in patients at intermediate-high or high risk of relapse [18].

Although ICIs have demonstrated remarkable clinical efficacy, their use is associated with a distinct spectrum of immune-related adverse events (irAEs) that can affect virtually any organ system, including nearly all endocrine glands [19–21]. These toxicities typically develop within the first 3–6 weeks of treatment but may occur at any time during therapy and even after its discontinuation [22,23]. Notably, endocrine dysfunction is permanent in roughly half of affected patients [19] and, if unrecognized or inadequately treated, can be life-threatening [21,24]. Delays in diagnosis are frequent and may necessitate the interruption or discontinuation of ICI therapy [21]. Additionally, ICI-based combination regimens are well-recognized to produce more frequent and higher-grade irAE compared to ICI monotherapy [25,26]. Similarly, VEGFR-TKIs such as axitinib, cabozantinib, and lenvatinib are associated with a broad range of adverse effects [27], including endocrine toxicity, particularly thyroid dysfunction and, most commonly, hypothyroidism [28]. Consequently, while VEGFR-TKIs + ICI combinations have improved anti-tumor efficacy, their use poses additional challenges, including the potential for additive toxicity and an increased risk of irAEs [29], particularly those involving the endocrine system [30]. Despite their widespread adoption, the relative contribution of VEGFR-TKIs and ICIs to endocrine irAEs remains poorly defined, and it is unclear whether combination therapy confers a higher, lower, or similar risk of endocrine toxicity compared with either agent alone.

The Food and Drug Administration (FDA) Adverse Event Reporting System (FAERS) is a publicly accessible database for post-marketing surveillance of FDA-approved therapies. It collects manufacturer- or consumer-initiated reports of AEs, product quality complaints, and medication errors, which are coded using the Medical Dictionary for Regulatory Activities (MedDRA) terminology. Although FAERS has inherent limitations, real-world data derived from this source can provide valuable insights and complement evidence from randomized controlled trials (RCTs) [31]. The primary aim of this study was to evaluate and compare the disproportionality of reported endocrine irAEs associated with VEGFR-TKI + ICI combinations approved for mRCC, vs. their corresponding VEGFR-TKI or ICI monotherapies (i.e., AP vs. axitinib [A], CN vs. cabozantinib [C], LP vs. lenvatinib [L], AP vs. P, CN vs. nivolumab [N], and LP vs. pembrolizumab [P]), irrespective of treatment line and setting, based on reports retrieved from the FAERS database. Deliberately, we did not include in our search the combination of two ICIs, ipilimumab and nivolumab, another first-line treatment standard of care. Indeed, the association of dual ICI therapy with a substantially higher incidence of endocrine irAEs compared with ICI monotherapy has already been demonstrated in pharmacovigilance analyses of the FAERS database [32–36]. Including these regimens would have considerably expanded the scope of the present study.

## 2 Materials and Methods

OpenVigil 2.1-MedDRA-v24 and AERS*Mine* [37] (data 2019 Q1–2024 Q2) were used as complementary tools for FAERS data querying and verification. Specifically, AERS*Mine* supported data exploration and consistency checks of drug–event associations, while OpenVigil 2.1 was primarily employed for structured disproportionality analyses. The 2019–2024 time frame was selected because, from 2019 onward, nearly all the drugs and combinations analyzed had been approved, thereby minimizing potential bias related to differences in time on the market. The number of total endocrine irAEs was extracted for VEGFR-TKI + ICI combinations (axitinib + pembrolizumab [AP], cabozantinib + nivolumab [CN], or lenvatinib + pembrolizumab [LP]), VEGFR-TKI monotherapy (axitinib [A], cabozantinib [C], or lenvatinib [L]), and ICI monotherapy (pembrolizumab [P] or nivolumab [N]). Then, in agreement with the MedDRA terminology, reports of endocrine irAEs identified by the preferred terms (PT) under the following high level terms (HLT) were retrieved: “adrenal cortical hyperfunctions”, “adrenal cortical hypofunction”, “adrenal gland disorder nec”, “adrenal medulla hyperfunction”, “adrenal neoplasms”, “diabetes mellitus incl subtypes”, hyperglycaemic conditions nec”, “hypoglycaemic conditions nec”, “female gonadal function disorders”, “male gonadal function disorders”, “endocrine abnormalities of gonadal function nec”, “anterior pituitary hyperfunction”, “anterior pituitary hypofunction”, “hypothalamus and pituitary gland disorders nec”, “posterior pituitary disorders”, “pituitary neoplasms”, “hyperparathyroid disorders”, “hypoparathyroid disorders”, “parathyroid disorders nec”, “thyroid disorders nec”, “thyroid hyperfunction disorders”, “thyroid hypofunction disorders”, “thyroid neoplasms”, “acute and chronic thyroiditis”, “ectopic endocrine disorders”, “endocrine neoplasms nec”, “multiple endocrine neoplasia syndromes”, “paraendocrine neoplasms nec” (Table S1). The overall analysis included 346 preferred terms (PT) divided in each high-level term (HLT). For each report, the presence of hospitalization, based on PT classification, was also recorded. For each PT, FAERS database provides indicators of severe adverse events (e.g., hospitalization, life-threatening events, disability, congenital anomalies, required intervention, death). In the present study, hospitalization was selected as a proxy for severity because it represents one of the most frequently reported seriousness indicators.

Automatic data deduplication is handled internally by the software using case identifiers and Individual Safety Report (ISR) numbers. AERS*Mine* adheres to FDA guidelines for duplicate removal by integrating ISRs, Case IDs, and version numbers to identify redundant entries or follow-up reports. Although raw FAERS data may contain duplicates, including multiple submissions for the same patient across different reporting quarters, AERS*Mine* automatically detects and excludes these instances. As a result, the analyses are unlikely to be substantially affected by potential over-reporting due to duplicated records. To account for multiple comparisons inherent to PT-level pharmacovigilance analyses, a false discovery rate (FDR) correction was applied. Specifically, results were filtered using the Benjamini–Hochberg procedure, as implemented within the FAERS analytical platform. This approach was used to limit the proportion of false-positive signals arising from multiple hypotheses. The proportional reporting ratio (PRR) and the reporting odds ratio (ROR) were extracted to assess the disproportionality reporting between cases and non-cases. Both increased and decreased disproportionality were considered, defined respectively by ROR or PRR values above or below 1, with 95% confidence interval excluding 1. Disproportionality analysis allows the detection of a higher reporting frequency of a certain adverse event with the investigated drug (or combination of drugs) in comparison with a reference represented by another specific drug (or combination of drugs) [38].

The OpenVigil 2 × 2 contingency table (https://openvigil.pharmacology.uni-kiel.de/contingency-table-calculator.php) was used to assess differences in the reporting of endocrine irAEs among users of VEGFR-TKI + ICI combinations compared with either the corresponding VEGFR-TKI monotherapy (“axitinib + pembrolizumab” vs. “axitinib”; “cabozantinib + nivolumab” vs. “cabozantinib”; “lenvatinib + pembrolizumab” vs. “lenvatinib”) or the corresponding ICI monotherapy (“axitinib + pembrolizumab” vs. “pembrolizumab”; “cabozantinib + nivolumab” vs. “nivolumab”; “lenvatinib + pembrolizumab” vs. “pembrolizumab”), retrieving chi-squared values, PRR, and ROR. Given the presence of sparse data and low expected frequencies for several drug–event combinations, Yates’ continuity correction was applied to chi-square testing to provide more conservative estimates and limit false-positive signals. The search was further refined by excluding patients who, in addition to the drug or combination therapy to which they were assigned, had received any of the other drugs included in this study during the course of their disease. The analysis was also performed separately for hospitalization as a proxy for severity. Figures were created with RStudio 2024.09.0+375, R package ggplot 2 version 3.4.1.

## 3 Results

VEGFR-TKI + ICI was associated with a total of 4267 reports of endocrine irAEs, accounting for 15.14% of all reported irAEs. These reports were related to 48 PT (Table S2). Among these, based on the HLT classification, 5 PT (23.2%) involved endocrine irAEs concerned adrenal gland disorders, 12 (25%) hypothalamus and pituitary gland disorders, 20 (41.67%) thyroid gland disorders, 1 (2.08%) parathyroid gland disorders, 9 (18.75%) glucose metabolism disorders, and 1 (2.08%) neoplastic and ectopic endocrinopathies (Table S3).

VEGFR-TKI monotherapy was associated with a total of 5624 reports of endocrine irAEs, accounting for 6.77% of all reported irAEs. These reports were related to 57 PT (Table S2). Specifically, based on the HLT classification, 3 PT (5.26%) endocrine irAEs concerned adrenal gland disorders, 14 (24.56%) hypothalamus and pituitary gland disorders, 27 (47.37%) thyroid gland disorders, 1 (1.75%) parathyroid gland disorders, 10 (17.54%) glucose metabolism disorders and 2 (3.51%) neoplastic and ectopic endocrinopathies (Table S4).

ICI monotherapy was associated with 22,349 reports of endocrine irAEs, accounting for 14.67% of all reported irAEs. These reports were related to 80 PT (Table S2). In details, based on the HLT classification, 5 PT (6.25%) involved endocrine irAEs concerned adrenal gland disorders, 17 (21.25%) hypothalamus and pituitary gland disorders, 30 (37.5%) thyroid gland disorders, 4 (5%) parathyroid gland disorders, 16 (20%) glucose metabolism disorders, 4 (5%) endocrine disorders of gonadal function and 4 (5%) neoplastic and ectopic endocrinopathies (Table S5).

### 3.1 VEGFR-TKI + ICI vs. VEGFR-TKI Monotherapy

In the overall population, several endocrine irAEs were disproportionately reported with VEGFR-TKI + ICI treatment compared with VEGFR-TKI monotherapy. The combination was associated with a higher reporting of endocrine irAEs, although the magnitude of association varied across the different endocrine axes, as detailed in the following sections (Table 1). When available, no consistent differences emerged in the proportion of severe reports across treatment groups (Table S6). Additionally, no significant disproportionate reporting was observed for all PTs concerning “endocrine disorders of gonadal function”, “parathyroid gland disorders dysfunction”, and “neoplastic and ectopic endocrinopathies”.

**Table 1:** Reporting odds ratio of endocrine adverse events for VEGFR-TKI + ICI vs. VEGFR-TKI monotherapy.

SOC: Endocrine Disorders												
	**VEGFR-TKI + ICI vs. VEGFR-TKI**											
	**AP vs. A**				**CN vs. C**				**LP vs. L**			
	**ROR**	**LCI**	**HCI**	***p***	**ROR**	**LCI**	**HCI**	***p***	**ROR**	**LCI**	**HCI**	***p***
**HLGT: ADRENAL GLAND DISORDERS**												
**HLT:** **Adrenal cortical hypofunction**												
**Adrenal insufficiency**	2.92	1.83	4.67	<0.01	9.35	6.97	12.56	<0.01	21.35	15.25	29.90	<0.01
**HLT:** **Adrenal gland disorders nec**												
**Adrenal disorder**	n.a.				7.42	3.60	15.28	<0.01	n.a.			
**HLT:** **Adrenal neoplasms**												
**Metastases to adrenals**	n.a.				5.15	2.19	12.14	<0.01	n.a.			
**HLGT: HYPOTHALAMUS AND PITUITARY GLAND DISORDERS**												
**HLT:** **Anterior pituitary hypofunction**												
**Hypopituitarism**	2.48	1.07	5.72	<0.05	3.15	1.45	6.82	<0.01	1.67	1.16	2.42	<0.01
**Adrenocorticotropic hormone deficiency**	3.85	1.11	13.30	<0.05	4.96	1.81	13.68	<0.01	1.70	1.10	2.63	<0.05
**Thyroid stimulating hormone deficiency**	n.s.				5.66	1.14	28.06	<0.05	n.a.			
**HLT:** **Hypothalamus and pituitary gland disorders nec**												
**Hypophysitis**	2.89	1.00	8.32	<0.05	3.72	2.10	6.63	<0.01	1.74	1.11	2.71	<0.05
**Hypothalamo-pituitary disorder**	3.03	1.37	6.67	<0.01	n.a.				n.s.			
**HLGT: Thyroid Gland Disorders**												
**HLT: Thyroid disorders nec**												
**Thyroid disorder**	1.58	1.09	2.28	<0.05	2.50	1.98	3.16	<0.01	3.26	2.64	4.02	<0.01
**HLT: Thyroid hyperfunction disorders**												
**Hyperthyroidism**	1.88	1.25	2.82	<0.01	2.67	2.09	3.42	<0.01	1.52	1.28	1.79	<0.01
**Immune-mediated hyperthyroidism**	3.85	1.24	11.94	<0.05	5.66	1.14	28.07	<0.05	1.83	1.07	3.12	<0.05
**Basedow’s disease**	3.85	1.44	10.69	<0.01	n.a				n.a			
**Graves’ disease**	n.s.				5.66	1.64	19.58	<0.01	n.a			
**HLT: Thyroid hypofunction disorders**												
**Hypothyroidism**	1.61	1.27	2.03	<0.01	1.51	1.29	1.78	<0.01	1.49	1.38	1.61	<0.01
**Immune-mediated hypothyroidism**	3.86	1.88	7.90	<0.01	3.40	1.23	9.36	<0.05	1.84	1.51	2.24	<0.01
**HLT: Acute and chronic thyroiditis**												
**Thyroiditis**	1.87	1.03	3.40	<0.05	3.83	2.35	6.25	<0.01	1.54	1.26	1.88	<0.01
**Immune-mediated thyroiditis**	n.s				5.67	2.25	14.28	<0.01	n.s			
**Thyroiditis subacute**	n.a				3.78	1.07	13.38	<0.05	n.s			
**Autoimmune thyroiditis**	n.a				4.05	1.28	12.75	<0.05	n.a			
**HLGT: Glucose Metabolism Disorders**												
**HLT:** **Diabetes mellitus incl subtypes**												
**Type 1 diabetes mellitus**	n.s.				4.10	2.01	8.36	<0.01	1.79	1.34	2.40	<0.01
**Fulminant type 1 diabetes mellitus**	n.a.				5.67	2.13	15.10	<0.01	n.s			
**HLT:** **Hyperglicaemic conditions nec**												
**Hyperglycaemia**	n.s.				3.76	2.53	5.59	<0.01	1.50	1.05	2.14	<0.05
**HLT:** **Hypoglycaemic conditions nec**												
**Hypoglycaemia**	2.36	1.33	4.19	<0.01	2.13	1.40	3.24	<0.01	n.s.			

Note: SOC: System Organ Class. HLGT: High Level Group Term. HLT: High Level Term; AP: axitinib + pembrolizumab; A: axitinib. CN: cabozantinib + nivolumab; C: cabozantinib; LP: lenvatinib + pembrolizumab. L: Lenvatinib; ROR: reporting odds ratio. LCI: low confidence interval; HCI: high confidence interval; n.a.: not applicable (0 adverse events recorded for one of the two comparators); n.s.: not significant.

#### 3.1.1 Adrenal Gland Disorders

**Figure 1:**
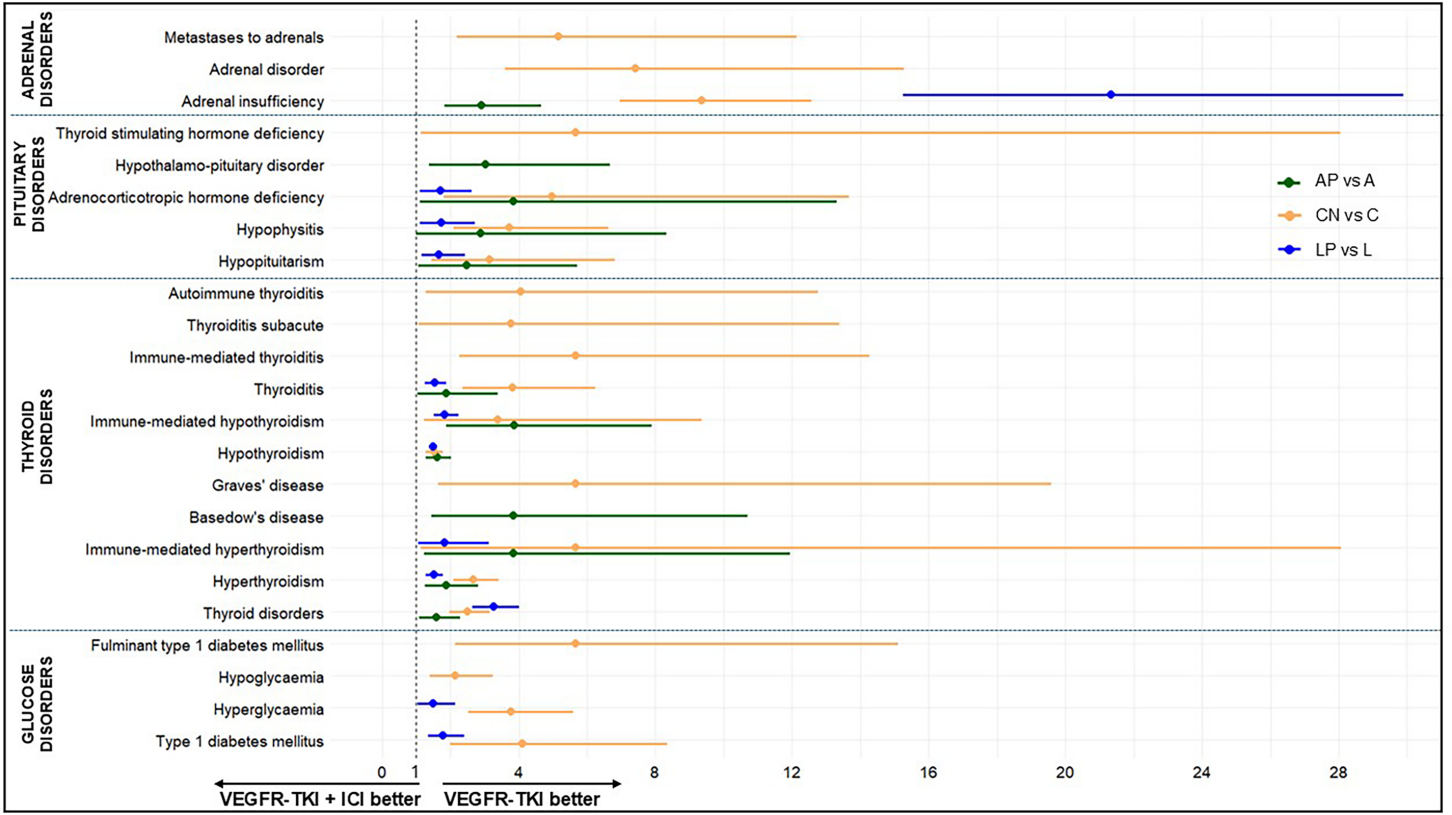
Endocrine immune related adverse events (irAEs) of interest with significant disproportional reporting for Vascular Endothelial Growth Factor Receptor Tyrosine Kinase Inhibitor (VEGFR-TKI) + Immune Checkpoint Inhibitor (ICI) vs. VEGFR-TKI monotherapy in the FAERS database. The forest plot shows reporting odds ratios (ROR) with 95% confidence intervals for adrenal gland disorders, hypothalamus and pituitary gland disorders, thyroid gland disorders, and glucose metabolism disorders for VEGFR-TKI + ICI vs. VEGFR-TKI monotherapy. Note: A: axitinib; AP: axitinib + pembrolizumab; C: cabozantinib; CN: cabozantinib + nivolumab; ICI: immune check-point inhibitors; L: lenvatinib; LP: lenvatinib + pembrolizumab; VEGFR-TKIs: vascular endothelial growth factor-tyrosine kinase inhibitor.

Disproportionate reporting of several adrenal disorders was detected, likely related to the administration of VEGFR-TKI + ICI vs. VEGFR-TKI monotherapy. The strongest and most consistent signals were observed for “adrenal insufficiency” (AP vs. A ROR: 2.92, 95% CI: 1.83–4.67, *p* < 0.01; CN vs. C ROR: 9.35, 95% CI: 6.97–12.56, *p* < 0.01, LP vs. L ROR: 21.35, 95% CI: 15.25–29.90, *p* < 0.01). Milder signals were also detected for “adrenal disorders” and “metastases to adrenal” in the CN vs. C comparison (Table 1 and Fig. 1). The strength of the association was similar among regimens. When available, no consistent differences emerged in the proportion of severe reports across treatment groups (Table S6).

#### 3.1.2 Hypothalamus and Pituitary Gland Disorders

Disproportionate reporting was observed for different hypothalamus and pituitary gland disorders, with significantly higher reporting among patients receiving VEGFR-TKI + ICI compared with VEGFR-TKI monotherapy. The strongest association was observed for “adrenocorticotropic hormone deficiency” (AP vs. A ROR: 3.85, 95% CI: 1.11–13.30, *p* < 0.05, CN vs. C ROR: 4.96, 95% CI: 1.81–13.68, *p* < 0.01, LP vs. L ROR: 1.70, 95% CI: 1.10–2.63, *p* < 0.05). Additional, albeit weaker, associations were identified for “thyroid stimulating hormone deficiency”, “hypothalamus and pituitary disorders”, and “hypophysitis” and “hypopituitarism” (Table 1 and Fig. 1). Severe events were significantly more frequently reported for “hypopituitarism” in the LP vs. L comparison and for “hypophysitis” in the CN vs. C and LP vs. L comparisons (Table S6).

#### 3.1.3 Thyroid and Parathyroid Gland Disorders

Disproportionate reporting signals were detected for multiple thyroid disorders, most notably “immune-mediated hyperthyroidism” in association with VEGFR-TKI + ICI compared with VEGFR-TKI monotherapy (AP vs. A ROR: 3.85, 95% CI: 1.24–11.94, *p* < 0.05, CN vs. C ROR: 5.66, 95% CI: 1.14–28.07, *p* < 0.05, LP vs. L ROR: 1.83, 95% CI: 1.07–3.12, *p* < 0.05). Weaker, yet still significant, signals were also observed for “thyroid disorders”, “hyperthyroidism”, “secondary hyperthyroidism”, “Basedow’s disease”, “Graves’ disease”, “hypothyroidism”, “immune-mediated hypothyroidism”, “thyroiditis”, “immune-mediated thyroiditis”, “thyroiditis subacute”, and “thyroiditis autoimmune” with different disproportionate signals among regimens (Table 1 and Fig. 1). When available, no consistent differences emerged in the proportion of severe reports across treatment groups (Table S6).

#### 3.1.4 Glucose Metabolism Disorders

Among glucose metabolism disorders, disproportionate reporting emerged for “fulminant type 1 diabetes”, “type 1 diabetes mellitus”, “hyperglycaemia”, and “hypoglycaemia”, seemingly associated with the administration of VEGFR-TKI + ICI compared with VEGFR-TKI monotherapy. Notably, higher signals were observed for “type 1 diabetes mellitus” (CN vs. C ROR: 4.10, 95% CI: 2.01–8.36, *p* < 0.01, LP vs. L ROR: 1.79, 95% CI: 1.34–2.40, *p* <0.01) (Table 1 and Fig. 1). When available, no consistent differences emerged in the proportion of severe reports across treatment groups (Table S6).

### 3.2 VEGFR-TKI + ICI vs. ICI Monotherapy

Within the overall population, the use of VEGFR-TKI + ICI was characterized by a disproportionate report for multiple endocrine irAEs compared with ICI monotherapy. These events showed variable association with either monotherapy or combination regimens, likely reflecting differences in the underlying pathophysiological mechanisms of the individual cases (Table 2). No consistent differences emerged in the proportion of severe reports across treatment groups, except for sporadic PTs (Table S7). Additionally, no significant disproportionate reporting was observed for all PTs concerning “endocrine disorders of gonadal function”, “parathyroid gland disorders dysfunction”, and “neoplastic and ectopic endocrinopathies”.

**Table 2:** Reporting odds ratio of endocrine adverse events for VEGFR-TKI + ICI vs. ICI monotherapy.

SOC: Endocrine Disorders												
	**VEGFR-TKI + ICI vs. ICI**											
	**AP vs. P**				**CN vs. N**				**LP vs. P**			
	**ROR**	**LCI**	**HCI**	***p***	**ROR**	**LCI**	**HCI**	***p***	**ROR**	**LCI**	**HCI**	***p***
**HLGT: Adrenal Gland Disorders**												
**HLT:** **Adrenal cortical hypofunction**												
**Adrenal insufficiency**	n.s.				n.s.				2.75	2.43	3.11	<0.01
**HLT:** **Adrenal gland disorders nec**												
**Adrenal disorder**	0.49	0.26	0.90	<0.05	n.s.				0.30	0.20	0.44	<0.01
**HLT:** **Adrenal neoplasms**												
**Metastases to adrenals**	n.a.				4.70	2.27	9.71	<0.01	n.a.			
**HLGT: Hypothalamus and Pituitary Gland Disorders**												
**HLT:** **Anterior pituitary hypofunction**												
**Hypopituitarism**	n.s.				0.19	0.10	0.35	<0.01	n.s.			
**Adrenocorticotropic hormone deficiency**	0.32	0.13	0.78	<0.05	0.19	0.09	0.39	<0.01	0.52	0.37	0.72	<0.01
**Thyroid stimulating hormone deficiency**	19.50	2.75	138.5	<0.05	n.s.				n.a.			
**HLT:** **Hypothalamus and pituitary gland disorders nec**												
**Hypophysitis**	0.30	0.17	0.85	<0.05	0.30	0.19	0.47	<0.01	0.49	0.35	0.69	<0.01
**Lymphocytic hypophysitis**	n.a.				0.32	0.10	0.99	<0.05	n.s.			
**Immune-mediated hypophysitis**	n.a.				0.16	0.04	0.64	<0.01	n.a.			
**Secondary adrenocortical insufficiency**	n.s.				n.a.				0.39	0.23	0.67	<0.01
**Hypothalamo-pituitary disorder**	n.s.				0.16	0.06	0.44	<0.01	0.28	0.15	0.52	<0.01
**HLGT: Thyroid Gland Disorders**												
**HLT:** **Thyroid disorders nec**												
**Thyroid disorder**	1.73	1.25	2.40	<0.01	2.69	2.17	3.34	<0.01	1.41	1.17	1.69	<0.01
**HLT:** **Thyroid hyperfunction disorders**												
**Thyrotoxic crisis**	n.s				n.a				1.84	1.03	3.26	<0.05
**Hyperthyroidism**	n.s				1.28	1.03	1.58	<0.05	1.39	1.21	1.61	<0.01
**Basedow’s disease**	5.79	2.63	12.75	<0.01	n.s				n.a			
**Graves’ disease**	11.15	3.27	38.11	<0.01	3.73	1.38	10.11	<0.01	n.a			
**HLT:** **Thyroid hypofunction disorders**												
**Hypothyroidism**	n.s				1.26	1.08	1.46	<0.01	2.23	2.07	2.39	<0.01
**Immune-mediated hypothyroidism**	0.55	0.33	0.91	<0.05	0.32	0.14	0.71	<0.01	1.49	1.26	1.75	<0.01
**Autoimmune hypothyroidism**	n.a				n.a				0.14	0.03	0.58	<0.01
**Myxoedema coma**	4.88	1.38	17.29	<0.05	n.a				n.a			
**HLT:** **Acute and chronic thyroiditis**												
**Thyroiditis**	n.s				0.56	0.38	0.82	<0.01	1.53	1.29	1.83	<0.01
**Immune-mediated thyroiditis**	n.s				n.s				0.37	0.22	0.65	<0.01
**Thyroiditis subacute**	n.a				n.s				2.82	1.35	5.91	<0.01
**Thyroiditis acute**	n.a				n.a				3.49	1.27	9.64	<0.05
**Autoimmune thyroiditis**	n.a				0.34	0.14	0.82	<0.05	n.a			
**HLGT: Glucose Metabolism Disorders**												
**HLT:** **Diabetes mellitus incl subtypes**												
**Diabetes mellitus**	n.s.				0.31	0.18	0.51	<0.01	0.49	0.37	0.65	<0.01
**Type 1 diabetes mellitus**	0.59	0.35	0.99	<0.05	0.26	0.15	0.46	<0.01	0.72	0.57	0.90	<0.01
**Fulminant type 1 diabetes mellitus**	n.a				0.12	0.06	0.24	<0.01	n.s.			
**HLT:** **Hypoglycaemic conditions nec**												
**Hypoglycaemia**	3.23	1.99	5.26	<0.01	1.90	1.30	2.80	<0.01	n.s.			

Note: N: nivolumab; P: pembrolizumab. n.a.: not applicable (0 adverse events recorded for one of the two comparators); n.s.: not significant.

#### 3.2.1 Adrenal Gland Disorders

**Figure 2:**
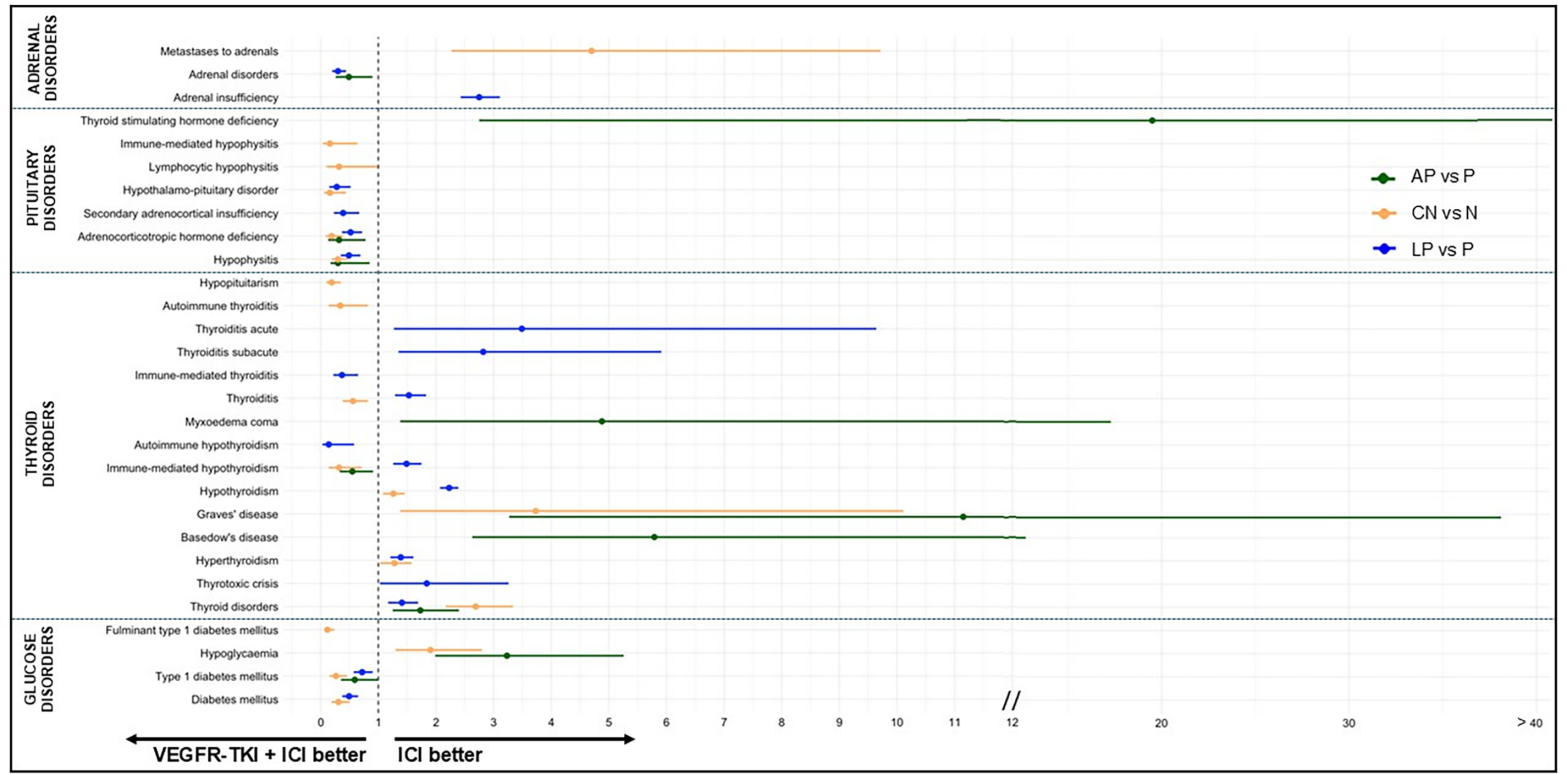
Endocrine irAEs of interest with significant disproportional reporting for VEGFR-TKI + ICI vs. ICI monotherapy in the FAERS database. The forest plot shows reporting odds ratios (ROR) with 95% confidence intervals for adrenal gland disorders, hypothalamus and pituitary gland disorders, thyroid gland disorders, and glucose metabolism disorders for VEGFR-TKI + ICI vs. ICI monotherapy. Note: N: nivolumab; P: pembrolizumab.

Disproportionate reporting of several adrenal disorders was detected. Specifically, “adrenal insufficiency” (LP vs. P ROR: 2.75, 95% CI: 2.43–3.11, *p* < 0.01) and “metastases to adrenals” (CN vs. N ROR: 4.70, 95% CI: 2.27–9.71, *p* < 0.01) were more frequently reported in association with the combination regimen. Conversely, “adrenal disorders” (AP vs. P ROR: 0.49, 95% CI: 0.26–0.90, *p* < 0.05; LP vs. P ROR: 0.30, 95% CI: 0.20–0.44, *p* < 0.01) were significantly more reported among patients receiving ICI monotherapy (Table 2 and Fig. 2), although more severe events were observed with the AP combination regimen compared to P monotherapy (Table S7).

#### 3.2.2 Hypothalamus and Pituitary Gland Disorders

Disproportionate reporting was observed for different hypothalamus and pituitary gland disorders, showing significantly higher reporting in patients receiving ICI monotherapy in most cases. The most robust and consistent associations were identified for “adrenocorticotropic hormone deficiency” (AP vs. P ROR: 0.32, 95% CI: 0.13–0.78, *p* < 0.05; CN vs. N ROR: 0.19, 95% CI: 0.09–0.39, *p* < 0.01; LP vs. P ROR: 0.52, 95% CI: 0.37–0.72, *p* < 0.01) and “hypophysitis” (AP vs. P ROR: 0.30, 95% CI: 0.17–0.85, *p* < 0.05; CN vs. N ROR: 0.30, 95% CI: 0.19–0.47, *p* < 0.01; LP vs. P ROR: 0.49, 95% CI: 0.35–0.69, *p* < 0.01). Additional, albeit weaker, associations were observed for “hypopituitarism”, “lymphocytic hypophysitis”, “immune-mediated hypophysitis”, “secondary adrenocortical insufficiency”, and “hypothalamus-pituitary disorders”. In contrast, “thyroid stimulating hormone deficiency” was more frequently reported in patients receiving combination regimens (Table 2 and Fig. 2). When available, no consistent differences emerged in the proportion of severe reports across treatment groups (Table S7).

#### 3.2.3 Thyroid and Parathyroid Gland Disorders

Disproportionate reporting signals were detected for multiple thyroid disorders, showing variable association with either ICI monotherapy or combination regimens. Specifically, “thyrotoxic crisis”, “hyperthyroidism”, “Basedow’s disease”, “Graves’ disease”, “hypothyroidism”, “myxoedema coma”, “thyroiditis subacute”, “thyroiditis acute”, and most consistently “thyroid disorders” (AP vs. P ROR: 1.73, 95% CI: 1.25–2.40, *p* < 0.01; CN vs. N ROR: 2.69, 95% CI: 2.17–3.34, *p* < 0.01; LP vs. P ROR: 1.41, 95% CI: 1.17–1.69, *p* < 0.01) were more frequently reported in patients receiving combination regimens. In contrast, “immune-mediated thyroiditis” and “autoimmune thyroiditis” were more frequently reported in patients receiving ICI monotherapy. Notably, “immune-mediated hypothyroidism” was more frequently reported with ICI monotherapy in the AP vs. P and CN vs. N comparisons, whereas it was more frequently reported with combination regimen in the LP vs. P comparison (AP vs. P ROR: 0.55, 95% CI: 0.33–0.91, *p* < 0.05; CN vs. N ROR: 0.32, 95% CI: 0.14–0.71, *p* < 0.01; LP vs. P ROR: 1.49, 95% CI: 1.26–1.75, *p* < 0.01). Similarly, “thyroiditis” was more frequently reported with monotherapy in the CN vs. N comparison (ROR: 0.56, 95% CI: 0.38–0.82, *p* < 0.01) and with combination regimen in the LP vs. P comparison (ROR: 1.53, 95% CI: 1.29–1.83, *p* < 0.01) (Table 2 and Fig. 2). When available, no consistent differences were observed in the proportion of severe reports across treatment groups, except for “immune-mediated hypothyroidism” in the AP vs. P comparison (ROR: 3.00, 95% CI: 1.19–7.58, *p* < 0.05) and “thyroiditis” in the CN vs. N comparison (ROR: 2.52, 95% CI: 1.66–5.43, *p* < 0.05), which showed higher proportions of severe events in the combination and monotherapy groups, respectively, contrary to the pattern observed for overall reports (Table S7).

#### 3.2.4 Glucose Metabolism Disorders

Among glucose metabolism disorders, disproportionate reporting emerged for “diabetes mellitus”, “fulminant type 1 diabetes mellitus”, and most consistently for “type 1 diabetes mellitus” (AP vs. P ROR: 0.59, 95% CI: 0.35–0.99, *p* < 0.05; CN vs. N ROR: 0.26, 95% CI: 0.15–0.46, *p* < 0.01; LP vs. P ROR: 0.72, 95% CI: 0.57–0.90, *p* < 0.01), appearing to be predominantly associated with ICI monotherapy (Table 2 and Fig. 2). In contrast, “hypoglycemia” was more frequently reported in patients receiving combination regimens. When available, no consistent differences emerged in the proportion of severe reports across treatment groups (Table S7).

## 4 Discussion

In this large pharmacovigilance analysis, we characterized the spectrum of endocrine irAEs associated with VEGFR-TKIs and ICIs, administered either as monotherapy or in combination, for the treatment of mRCC. Overall, our findings suggest that VEGFR-TKIs + ICI combinations, compared with VEGFR-TKI monotherapy, are associated with increased reporting of several endocrine irAEs. Conversely, when compared with ICI monotherapy, the combination showed a more heterogeneous disproportionality pattern, with generally lower reporting of hypothalamus, pituitary, and hyperglycemic disorders, alongside higher reporting of hypoglycemic and thyroid irAEs, with the exception of autoimmune thyroid diseases. Notably, the safety profile varied across specific drug regimens and endocrine axes.

### 4.1 Adrenal Gland Disorders

Our analysis revealed a higher disproportionality signal for adrenal gland disorders when VEGFR-TKI + ICI regimens were compared with VEGFR-TKI monotherapy. By contrast, comparison with ICI monotherapy showed heterogeneous patterns. Specifically, “adrenal disorder” was more frequently reported with monotherapy in the AP vs. P and LP vs. P comparisons; however, more severe events were observed with the AP combination regimen compared to P monotherapy, possibly reflecting its use in patients with more advanced disease. Additionally, “adrenal insufficiency” showed lower reporting with monotherapy in the LP vs. P comparison, and “metastases to adrenal” was less frequently reported with monotherapy in the CN vs. N comparison. Adrenal dysfunction, although relatively uncommon, is a well-established irAE in patients receiving ICIs [19–21], particularly nivolumab and pembrolizumab [39]. In contrast, the role of VEGFR-TKIs in adrenal toxicity remains poorly explored, with only sparse evidence available [28]. Clinical data mainly report subclinical adrenal insufficiency, particularly with lenvatinib [40–42]. These findings suggest that ICIs are the primary drivers of adrenal gland disorders, with a modest but non-negligible contribution from VEGFR-TKIs. This likely explains the higher reporting of adrenal adverse events observed with combination regimens compared with VEGFR-TKI monotherapy, as well as the heterogeneous results observed when combinations are compared with ICI monotherapy, where disproportionality may depend on the specific VEGFR-TKI used and the magnitude of its additive effect with ICIs. The precise mechanism underlying ICI-induced adrenocortical insufficiency remains unclear, but it is realistically autoimmune in nature. Blockade of CTLA-4 or PD-1/PD-L1 may promote adrenal inflammation, and adrenal autoantibodies (e.g., anti-21α-hydroxylase) have been reported in most cases of autoimmune adrenalitis, although causality remains uncertain [43]. For VEGFR-TKIs, mechanistic data are limited; some reports suggest that VEGF pathway inhibition may cause adrenal microvascular damage or ischemia, but systematic evidence is lacking [44].

### 4.2 Hypothalamus and Pituitary Gland Disorders

We also observed a higher reporting rate of pituitary disorders with VEGFR-TKI + ICI compared with VEGFR-TKI alone. In contrast, comparison with ICI monotherapy showed a reduced disproportionality signal for endocrine toxicity with the combination. These findings are consistent with strong clinical evidence showing that hypophysitis is a typical ICI-related toxicity, particularly with CTLA-4 blockade, but also with PD-1 inhibitors such as nivolumab and pembrolizumab [19,45]. In contrast, pituitary disorders related to VEGFR-TKIs therapy remain controversial, and no association with hypophysitis has been described [28]. The increased reporting of hypophysitis observed for VEGFR-TKI + ICI combination is most likely explained by the contribution of ICI, as pituitary inflammation is a well-recognized irAE associated with PD-1 blockade [21]. VEGFR-TKIs-related pituitary disorders are less clearly defined. Some VEGFR-TKIs, including axitinib, may interfere with the hypothalamic–pituitary–thyroid axis, altering TSH regulation [46,47]. The only contrasting finding was observed for “thyroid-stimulating hormone deficiency” in the AP vs. P comparison; however, this result should be interpreted with caution, as it may be driven by the very small number of reports for this PT (only two reports in the AP group and two in the P group).

### 4.3 Thyroid and Parathyroid Gland Disorders

Our results showed increased reporting of several thyroid disorders, particularly those with a destructive pathogenesis, with VEGFR-TKI + ICI combinations compared with either VEGFR-TKI or ICI monotherapy, whereas the reporting frequency of autoimmune thyroid disorders in VEGFR-TKI + ICI combinations was reduced compared with ICI monotherapy. These findings are consistent with existing evidence [19]. Thyroid dysfunction is among the most frequent irAEs associated with ICIs, particularly frequent with PD-1/PD-L1 inhibitors [48]. A recent systematic review and meta-analysis [49] confirmed a high reporting frequency for thyroid dysfunction with PD-1/PD-L1 inhibitors, especially hypothyroidism (pembrolizumab 8.5%, nivolumab 8.0%, PD-L1 inhibitors 5.5%). Hyperthyroidism is less common, occurring in 1%–4% of cases, often as a transient phase preceding hypothyroidism [19]. Although incidence varies depending on the agent, thyroid dysfunction is also a frequent toxicity of VEGFR-TKIs [28], which may counterbalance the mitigating effect observed for other endocrine dysfunction, such as hypothalamus and pituitary gland disorders, and glucose metabolism disturbances. ICI-induced thyroid disorders are thought to result from T-cell–mediated autoimmunity, often preceded by transient thyrotoxicosis due to destructive thyroiditis. Proposed mechanisms include proliferation of follicular helper T cells, production of thyroid autoantibodies, HLA-DR overexpression, macrophage activation, and cytokine-driven cytotoxic T-cell activity [48,50]. PD-1/PD-L1 blockade appears particularly implicated in triggering thyroid autoimmunity [48]. In contrast, VEGFR-TKIs-induced thyroid dysfunction is believed to result primarily from VEGF receptor (VEGFR) and platelet-derived growth factor receptors (PDGFR) inhibition, leading to thyroid microvascular injury and destructive thyroiditis. Additional mechanisms may include thyroid peroxidase (TPO) inactivation, impaired iodine uptake, and altered TRH regulation. This difference in pathogenetic mechanism explains both the higher disproportionality observed for destructive thyroiditis and the lower disproportionality for autoimmune thyroid disorders with combination therapy compared with ICI monotherapy. Notably, “immune-mediated hypothyroidism” was more frequently reported with ICI monotherapy in the AP vs. P and CN vs. N comparisons, whereas it was more frequently reported with the combination regimen in the LP vs. P comparison. This difference may be attributable to the fact that, among VEGFR-TKI agents, lenvatinib is the most strongly associated with thyroid dysfunction [28].

### 4.4 Glucose Metabolism Disorders

Finally, glucose metabolism disorders were more frequently reported with VEGFR-TKI + ICI compared with VEGFR-TKI monotherapy, but less frequently compared with ICI monotherapy. This pattern aligns with prior evidence showing that ICIs, including PD-L1 inhibitors, can trigger autoimmune diabetes, with recent case series reporting rates between 0.2% and 1.8% [21]. All currently approved ICIs have been associated with incident diabetes, especially PD-1/PD-L1 inhibitors [19]. VEGFR-TKIs have also been linked to metabolic disturbances, although evidence remains heterogeneous [28]. An exception to this overall trend was observed for “hypoglycaemia”, which showed higher reporting with combination regimens compared with both VEGFR-TKI and ICI monotherapies. Notably, unlike ICIs, which are primarily associated with hyperglycemia, VEGFR-TKIs can induce both hyperglycemia and hypoglycemia depending on the specific agent. For example, axitinib has been reported to cause hypoglycemia in up to 11%–24% of patients. Proposed mechanisms include inhibition of IGF-1 and insulin receptor kinases, increased insulin resistance, and impaired β-cell function [48]. These observations suggest that VEGFR-TKIs are the main contributors to hypoglycemia, which may explain the higher reporting observed with combination regimens compared with ICI monotherapy. Conversely, the increased reporting of hypoglycemia with combination therapy compared with VEGFR-TKI monotherapy could reflect the preferential use of combination regimens in patients with more advanced disease, who may be affected by cachexia.

### 4.5 Concluding remarks

Overall, our results showed that all major endocrine irAEs were reported more frequently with the combination of VEGFR-TKI and ICI than with VEGFR-TKIs monotherapy. This finding was expected, as endocrine toxicities are well known to be predominantly ICI-driven rather than VEGFR-TKIs-related, in line with published literature. Conversely, when compared with ICI monotherapy, the addition of a VEGFR-TKI appeared to reduce the reporting frequency of hypothalamus, pituitary, immune-mediated thyroid, and glucose metabolism disorders, except for a few sporadic cases, while no substantial mitigating effect was observed for adrenal disorders or destructive thyroiditis. These findings are consistent with a previous FAERS analysis exploring all irAEs, in which VEGFR-TKI + ICI therapy showed a globally reduced ROR for almost all immune-related AEs compared with ICI monotherapy [51].

From a pathophysiological perspective, ICIs promote T-cell activation against self-antigens, leading to immune-mediated destruction of endocrine tissues, most frequently the thyroid and pituitary glands [19]. VEGFR-TKIs, by inhibiting VEGF and related pathways, may contribute to vascular and microenvironmental changes that attenuate the immunogenicity of endocrine glands [1–3]. The rationale for combining ICIs with VEGFR-TKIs lies in the ability of anti-angiogenic therapy to remodel abnormal tumor vasculature and alleviate hypoxia, thereby restoring immune cell trafficking and enhancing anti-tumor immunity [1–3]. In neoplastic tissues, VEGF is frequently overexpressed due to tumor-driven hypoxia, and its inhibition can normalize tumor vasculature and improve the infiltration of cytotoxic T cells [1–3]. Conversely, in normal endocrine tissues where VEGF expression is not typically upregulated, exposure to anti-angiogenic agents may instead reduce vascular perfusion [27,28]. This could theoretically attenuate local immune cell infiltration and mitigate the immunogenicity of endocrine glands [27,28]. Supporting this hypothesis, Huang et al. demonstrated that high-dose of an experimental anti-VEGFR2 antibody aggravated hypoxia and suppressed CD8^+^ T-cell infiltration in tumor tissue, whereas lower doses normalized vessels and enhanced anti-tumor immunity [52]. A similar mechanism may occur in endocrine tissues exposed to VEGFR-TKIs, where excessive anti-angiogenic pressure could dampen immune activation and reduce the incidence of endocrine irAEs in VEGFR-TKI + ICI regimens [1–3]. Alternatively, this apparent protective effect could reflect differences in patient selection, treatment duration, or reporting practices, rather than a true biological interaction. Further mechanistic studies are warranted to clarify whether VEGFR-TKIs exert a mitigating effect on ICI-induced endocrine toxicity or whether this observation is mainly due to confounding factors [1–3]. Importantly, this protective effect is not observed in destructive thyroiditis and certain adrenal disorders, as these represent direct adverse events attributable to VEGFR-TKI therapy.

This study offers several notable strengths. Although adverse events associated with dual ICI therapy have been extensively investigated, only one recent pharmacovigilance study has broadly examined irAEs related to VEGFR-TKI + ICI combinations in FAERS. Our work extends this evidence in several important ways. First, we focused specifically on endocrine toxicities, providing an axis-by-axis assessment of adrenal, pituitary, thyroid, and glucose metabolism disorders rather than evaluating all irAEs collectively. Second, we performed dual comparisons (VEGFR-TKI + ICI vs. ICI and VEGFR-TKI + ICI vs. VEGFR-TKI), enabling us to disentangle the relative contribution of each drug class to endocrine adverse events. Third, we analyzed individual drug regimens approved for RCC, revealing molecule-specific differences with direct clinical relevance.

Fourth, we incorporate severity analyses, which strengthened the robustness and transparency of our findings while allowing us to highlight discrepancies between general and severe-restricted signals. Importantly, the use of the FAERS database, despite its inherent limitations, provides valuable real-world evidence that complements randomized clinical trials and observational cohorts by mitigating some selection biases. By examining specific drug–drug combinations and individual endocrine axes, our study offers a more granular perspective on the safety profiles of these regimens, generating new hypotheses and informing clinical practice.

Nevertheless, several limitations should be acknowledged, most of which are inherent to the FAERS database. FAERS relies on spontaneous adverse event reporting, which is affected by substantial underreporting and does not provide true incidence rates. Consequently, disproportionality metrics reflect reporting associations rather than causal relationships, and signals must be interpreted solely as hypothesis-generating. Moreover, FAERS accepts submissions from a wide range of reporters (healthcare professionals, patients, relatives, manufacturers, and other stakeholders), resulting in heterogeneous data quality, potential duplicates, and incomplete clinical information. Essential variables, such as treatment dosage and schedule, duration of exposure, indication, prior therapies, cancer stage, comorbidities, and laboratory confirmation of adverse events, are frequently missing or inconsistently documented, limiting reliable comparisons of endocrine irAEs across treatment groups. Importantly, FAERS does not provide standardized grading of event severity, precluding meaningful evaluation or comparison of the clinical impact of endocrine irAEs across therapies. Finally, reporting behavior can be strongly influenced by external factors, including public awareness of specific drug–event associations, media attention, geographic or ethnic differences, recent label changes, and the timing of drug approval and market penetration. To limit time-lag bias between different agents, the analysis was restricted in the range 2019–2024, when nearly all the drugs and combinations analyzed had been approved. The lack of reliable denominators (such as the number of exposed patients, prescription volume, or treatment duration) prevents adjustment for drug utilization patterns or population-level exposure, limiting direct comparison across regimens approved in different years. Limitations specific to our analysis include the focus on combination therapies approved for mRCC, which may not capture the broader spectrum of VEGFR-TKI + ICI use across other malignancies. For some events, Evan’s criteria were not fulfilled; in others, severity analyses failed to reach significance. These discrepancies likely reflect concomitant drug use in the overall dataset, differences in case attribution, reporting bias, and reduced statistical power in subgroup analyses, and should be interpreted with caution. Moreover, ROR values were calculated at the level of individual preferred terms and specific drug–drug comparisons, which limits the generalizability of our findings, as disproportionality signals may not uniformly apply across all agents within a therapeutic class. Although the FAERS Public Dashboard applies a Benjamini–Hochberg FDR filter during initial data extraction, no additional multiplicity adjustment was applied to ROR calculations. Therefore, some PT-level associations may still be affected by false-positive inflation due to multiple testing. Additionally, our disproportionality analysis relied on ROR and PRR, which are standard for FAERS signal detection; more advanced Bayesian approaches and time-trend analyses were not applied and could further improve signal robustness.

## 5 Conclusion

Overall, these findings suggest that, while the reporting of endocrine irAEs with VEGFR-TKI plus ICI combinations is consistently higher than with VEGFR-TKI monotherapy, it may be either lower or higher than with ICI monotherapy depending on the specific agent used and the affected endocrine site. This highlights the need for tailored monitoring and heightened vigilance in clinical practice. However, as this analysis is based on observational data from FAERS, it does not establish causality. Therefore, these results should be interpreted with caution and assessed on a case-by-case basis, while further studies are warranted to clarify any potential protective effect of VEGFR-TKIs.

## Supplementary Materials



## Data Availability

The authors confirm that the data supporting the findings of this study are available within the article and its Supplementary Materials.

## Supplementary Materials

The supplementary material is available online at https://www.techscience.com/doi/10.32604/or.2026.074672/s1.
