# Supplementary material for: The Real-World Endocrine Toxicity Profile of ICIs, VEGFR-TKIs, and Their Combination: Analysis of the FDA Adverse Event Reporting System (FAERS) Database
Source: Oncol Res. 2026 Apr 22;34(5):19. doi: 10.32604/or.2026.074672 (PMC13126590; doi:10.32604/or.2026.074672)
Supplement: Supplementary file 1 [file OncolRes-34-74672-s001.docx]

**The real‑world endocrine toxicity profile of immune checkpoint inhibitors (ICIs), vascular endothelial growth factor receptor tyrosine kinase inhibitors (VEGFR-TKIs), and their combination: Pharmacovigilance analysis of the FDA Adverse Event Reporting System (FAERS) database.**

**Supplementary files**

| Supplementary Table 1. Search strategy | Pag. 1 |
| --- | --- |
| Supplementary Table 2. Reports distribution based on PT classification | Pag. 6 |
| Supplementary Table 3. Adverse events with VEGFR-TKI + ICI therapy grouped according to High Level Group Term | Pag. 11 |
| Supplementary Table 4. Adverse events with VEGFR-TKI monotherapy grouped according to High Level Group Term | Pag. 11 |
| Supplementary Table 5. Adverse events with ICI monotherapy grouped according to High Level Group Term | Pag. 12 |
| Supplementary Table 6. Reporting odds ratio of endocrine adverse events requiring hospitalization for VEGFR-TKI + ICI vs. VEGFR-TKI monotherapy | Pag. 13 |
| Supplementary Table 7. Reporting odds ratio of endocrine adverse events requiring hospitalization for VEGFR-TKI + ICI vs. ICI monotherapy | Pag. 15 |

**Table S1.** Search strategy.

| **ENDOCRINE DISORDERS** | | | | | |  | | |  |
| --- | --- | --- | --- | --- | --- | --- | --- | --- | --- |
| **ADRENAL GLAND DISORDERS** | | | | | | | | | |
| ***Adrenal cortical hyperfunctions*** | ***Adrenal cortical hypofunction*** | | | ***Adrenal gland disorders nec*** | | ***Adrenal medulla hyperfunction*** | | | ***Adrenal neoplasms*** |
| Cushing's syndrome  cushingoid  hyperadrenalism  hypercorticoidism  hyperadrenocorticism  hyperaldosteronism  Nelson's syndrome  primary hyperaldosteronism  adrenal androgen excess  carney complex  apparent mineralocorticoid excess  acquired apparent mineralocorticoid excess  pseudoaldosteronism  secondary aldosteronism | Addison's disease  adrenocortical insufficiency chronic  adrenal atrophy  adrenal cortex atrophy  adrenal insufficiency  adrenal cortical insufficiency  adrenal suppression  adrenocortical insufficiency acute  hypoaldosteronism  steroid withdrawal syndrome  primary adrenal insufficiency  glucocorticoid deficiency  cortisol deficiency  mineralocorticoid deficiency  adrenal androgen deficiency  immune-mediated adrenal insufficiency  adrenogenital syndrome  triple a syndrome  pseudohypoaldosteronism | | | adrenalitis  adrenal cortex dysplasia  adrenal cortex necrosis  adrenal disorder  adrenal gland injury  adrenal haemorrhage  adrenal gland abscess  adrenal haematoma  adrenal gland tuberculosis  adrenal glomerular zone abnormal  hyperplasia adrenal  adrenoleukodystrophy  adrenomegaly  adrenal mass  adrenal calcification  adrenal thrombosis  hemorrhagic adrenal infarction  catecholamine crisis | | adrenal medulla hyperfunction  pseudophaeochromocytoma | | | adrenal adenoma  adrenocortical carcinoma  benign neoplasm of adrenal gland  metastases to adrenals  phaeochromocytoma  adrenal cyst  phaeochromocytoma malignant  adrenal gland cancer metastatic  adrenal neoplasms  phaeochromocytoma crisis  myelolipoma  adrenal carcinoma |
| **GLUCOSE METABOLISM DISORDERS** | | | | | | | | | |
| ***Diabetes mellitus incl subtypes*** | | | ***Hyperglycaemic conditions nec*** | | | | ***Hypoglycemic conditions nec*** | | |
| diabetes mellitus  diabetes complicating pregnancy  diabetes mellitus inadequate control  gestational diabetes  diabetes with hyperosmolarity  decreased insulin requirement  insulin-requiring type 2 diabetes mellitus  insulin-requiring type II diabetes mellitus  congenital generalised lipodystrophy  latent autoimmune diabetes in adults  cystic fibrosis related diabetes  fulminant type 1 diabetes mellitus  type 3 diabetes mellitus  monogenic diabetes  wolfram syndrome  steroid diabetes  new onset diabetes after transplantation  maternally inherited diabetes and deafness  type 1 diabetes mellitus  diabetes mellitus insulin-dependent  type 2 diabetes mellitus  diabetes mellitus non-insulin-dependent | | | glucose tolerance impaired  glucose tolerance impaired in pregnancy  glycosuria during pregnancy  hyperglycaemia  hypoinsulinaemia postoperative  insulin resistance  metabolic syndrome  insulin resistance syndrome  cardiometabolic syndrome  impaired insulin secretion  impaired fasting glucose  dawn phenomenon  hypoinsulinaemia  hyperglycaemic unconsciousness  hyperglycaemic seizure  hypoinsulinism  neonatal hyperglycaemia | | | | hypoglycaemia  hypoglycaemia neonatal  hypoglycaemia unawareness  hypoglycaemic coma  hypoglycaemic encephalopathy  shock hypoglycaemic  hypoglycaemic seizure  neuroglycopenia  postprandial hypoglycaemia  hyperinsulinaemia  hyperinsulinism  hypoglycaemic unconsciousness  pseudohypoglycaemia  hyperinsulinaemic hypoglycaemia  nesidioblastosis  paraneoplastic hypoglycaemia  congenital hyperinsulinaemic hypoglycaemia  hyperinsulinism syndrome | | |
| **ENDOCRINE DISORDERS OF GONADAL FUNCTION** | | | | | | | | | |
| ***Female gonadal function disorders*** | | | ***Male gonadal function disorders*** | | | | ***Endocrine abnormalities of gonadal function nec*** | | |
| amenorrhoea  anovulatory cycle  bleeding anovulatory  hirsutism  hypogonadism female  hypomenorrhoea  menstruation delayed  menstruation irregular  oligomenorrhoea  ovarian atrophy  ovarian failure postoperative  ovarian failure  ovarian hyperfunction  ovarian hyperstimulation syndrome  ovulation delayed  polycystic ovarian syndrome  premature menopause  enlarge clitoris  virilism  virilism foetal  hyperthecosis  ovulation disorder  hyperreactio luteinalis  ovarian hypoplasia  high response to ovarian stimulation | | | androgen deficiency  gynaecomastia  hypogonadism male  testicular atrophy  testicular failure  testicular failure primary  testicular hyperfunction  male genital atrophy  late onset hypogonadism syndrome  anorchism  cryptorchism  eunuchoidism  Klinefelter's syndrome  xxxy syndrome  xxyy syndrome  feminisation acquired  androgen insensitivity syndrome  sertoli-cell-only syndrome  bulbospinal muscular atrophy congenital  microorchidism | | | | gonadotrophin deficiency  hyperoestrogenism  oestrogen deficiency  hypogonadism  hypergonadism  primary hypogonadism  fertility increased  hermaphroditism  pseudohermaphroditism  pseudohermaphroditism female  pseudohermaphroditism male  Turner's syndrome  secondary sexual characteristics absence  congenital androgen deficiency  hyperandrogenism  oestrogenic affect  estrogenic effect  androgenetic alopecia  female pattern baldness  male pattern baldness  genital atrophy  hyperprogesteronism  hypoprogesteronism  persistent mullerian duct syndrome  post 5-α-reductase inhibitor syndrome  swyer syndrome | | |
| **HYPOTALAMUS AND PITUITARY GLAND DISORDERS** | | | | | | | | | |
| ***Anterior pituitary hyperfunction*** | ***Anterior pituitary hypofunction*** | | | ***Hypothalamus and pituitary gland disorders nec*** | | ***Posterior pituitary disorders*** | | | ***Pituitary neoplasms*** |
| acromegaly  gigantism  growth accelerated  hyperpituitarism  hyperprolactinaemia  pituitary-dependent Cushing's syndrome  pituitary gonadotropin hyperfunction  acral overgrowth  inappropriate thyroid stimulating hormone secretion  macroprolactinaemia | dwarfism  hypopituitarism  hypoprolactinemia  luteal phase deficiency  growth hormone deficiency  luteinising hormone deficiency  growth failure  adrenocorticotropic hormone deficiency  thyroid stimulating hormone deficiency  isolated adrenocorticotropic hormone deficiency  pituitary amenorrhoea  apituitarism | | | empty sella syndrome  galactorrhoea  hypothalamo-pituitary disorder  pituitary infarction  secondary adrenocortical insufficiency  pituitary enlargement  pituitary haemorrhage  pituitary hypoplasia  pituitary apoplexy  secondary hypogonadism  hypophysitis  lymphocytic hypophysitis  gonadotrophin releasing hormone deficiency  pituitary hyperplasia  hypothalamic pituitary adrenal axis suppression  growth disorder  immune-mediated hypophysitis | | antidiuretic hormone abnormality  diabetes insipidus  polydipsia  inappropriate antidiuretic hormone secretion | | | ACTH-producing pituitary tumour  growth hormone-producing pituitary tumour  malignant pituitary tumour  non-secretory adenoma of pituitary  pituitary tumour  prolactinoma  secretory adenoma of pituitary  thyroid stimulating hormone-producing pituitary tumour  metastases to pituitary gland  pituitary cyst  pituitary cancer metastases  pituitary tumour recurrent  pituitary neoplasm malignant recurrent  pituitary tumour benign |
| **Parathyroid gland disorders** | | | | | | | | | |
| ***Hypoparathyroid disorders*** | | | | | ***Parathyroid disorders nec*** | | | | |
| calcium deficiency  calcium intoxication  hypoparathyroidism  tetany  hypoparathyroidism secondary  primary hypoparathyroidism  Digeorge's syndrome  neonatal hypoparathyroidism  Barak syndrome  post procedural hypoparathyroidism | | | | | parathyroid disorder  hungry bone syndrome  parathyroid tumour  parathyroid tumour benign  parathyroid tumour malignant  parathyroid gland enlargement  parathyroid gland abscess  parathyroid haemorrhage  parathyroid cyst | | | | |
| **THYROID GLAND DISORDERS** | | | | | | | | | |
| ***Thyroid disorders nec*** | ***Thyroid hyperfunction disorders*** | | | ***Thyroid hypofunction disorders*** | | ***Thyroid neoplasms*** | | | ***Acute and chronic thyroiditis*** |
| euthyroid sick syndrome  goitre  thyroid disorder  thyroid mass  thyroid gland injury  autoimmune thyroid disorder  calcitonin secretion disorder  hypercalcitoninaemia  thyroid size decreased  goitre congenital  lid lag  thyroglossal cyst  thyroid pain  thyroid dysfunction in pregnancy  thyroid malformation  thyroglossal fistula  thyroid haemorrhage  thyroid fibrosis  ectopic thyroid  thyroid c-cell hyperplasia  haemorrhagic thyroid cyst  thyroid hemiagenesis  thyroid calcification | Basedow's disease  hyperthyroidism  thyrotoxic crisis  toxic nodular goitre  exophthalmos  secondary hyperthyroidism  endocrine ophthalmopathy  hashitoxicosis  toxic goitre  primary hyperthyroidism  immune-mediated hyperthyroidism  graves' disease  thyrotoxic periodic paralysis  congenital hyperthyroidism  Marine Lenhart syndrome  thyroid dermatopathy  thyrotoxic cardiomyopathy  thyrotoxic myopathy | | | generalised resistance to thyroid hormone  hypothyroidism  myxoedema  primary hypothyroidism  secondary hypothyroidism  tertiary hypothyroidism  thyroid atrophy  hypothyroidic goitre  myxoedema coma  autoimmune hypothyroidism  immune-mediated hypothyroidism  post procedural hypothyroidism  hypothyroidism postoperative  transient hypothyroxinaemia of prematurity | | anaplastic thyroid cancer  benign neoplasm of thyroid gland  follicular thyroid cancer  medullary thyroid cancer  papillary thyroid cancer  thyroid adenoma  thyroid cyst  thyroid neoplasm  metastases to thyroid  thyroid cancer metastatic  Hurthle cell carcinoma  thyroid cancer  thyroid cancer stage 0  thyroid cancer stage i  thyroid cancer stage ii  thyroid cancer stage iii  thyroid cancer stage iv  thyroid cancer recurrent  thyroid β-cell lymphoma  poorly differentiated thyroid carcinoma | | | thyroiditis  thyroiditis acute  thyroiditis chronic  thyroiditis fibrous chronic  thyroiditis subacute  autoimmune thyroiditis  atrophic thyroiditis  silent thyroiditis  immune-mediated thyroiditis  thyroid tuberculosis  thyroid gland abscess  thyroglossal cyst infection  radiation thyroiditis  Hashimoto's encephalopathy  infectious thyroiditis |
| **NEOPLASTIC AND ECTOPIC ENDOCRINOPATHIES** | | | | | | | | | |
| ***Ectopic endocrine disorders*** | | ***Endocrine neoplasms nec*** | | | ***Multiple endocrine neoplasia syndromes*** | | | ***Paraendocrine neoplasms nec*** | |
| carcinoid syndrome  ectopic ACTH syndrome  ectopic antidiuretic hormone secretion  ectopic calcitonin production  ectopic hyperthyroidism  ectopic parathyroid hormone production  ectopic parathormone production  hormone-secreting ovarian tumour  hypercalcaemia of malignancy  ectopic hormone secretion  carcinoid heart disease  carcinoid crisis | | benign neoplasm of islets of Langerhans  carcinoid tumour  carcinoid tumour of appendix  carcinoid tumour of the caecum  carcinoid tumour of the gastrointestinal tract  carcinoid tumour of the prostate  carcinoid tumour of the stomach  carcinoid tumour pulmonary  gastrinoma  glucagonoma  insulinoma  malignant neoplasm of islets of Langerhans  neuroendocrine carcinoma of the skin  vipoma  carcinoid tumour of the duodenum  gastrinoma malignant  neuroendocrine tumour  carcinoid tumour of the small bowel  carcinoid tumour of the pancreas  benign endocrine neoplasm  endocrine neoplasms NEC  endocrine neoplasm malignant  pancreatic neuroendocrine tumour  metastatic carcinoid tumour  pancreatic neuroendocrine tumour metastatic  neuroendocrine carcinoma metastatic  mixed adenoneuroendocrine carcinoma  gastroenteropancreatic neuroendocrine tumour disease  neuroendocrine tumour of the lung  neuroendocrine tumour of the lung metastatic  neuroendocrine carcinoma of the bladder  carcinoid tumour of the liver  carcinoid tumour of the ovary  neuroendocrine carcinoma of prostate  neuroendocrine tumour of the rectum  hepatic neuroendocrine tumour  small intestine neuroendocrine tumour | | | multiple endocrine neoplasia  multiple endocrine adenomatosis  multiple endocrine neoplasia type 2a  multiple endocrine neoplasia type 2  multiple endocrine neoplasia type II  multiple endocrine neoplasia type 1  multiple endocrine adenomatosis type I | | | paraganglion neoplasm benign  paraganglion neoplasm malignant  paraganglion neoplasm  carotid body tumour  chemodectoma  glomus jugulare tumour  glomus tympanicum tumour | |

System Organ Class (SOC) is indicated in bold capital letters; High Level Group Terms (HLGT) are indicated in capital letters; High Level Terms (HLT) are indicated in italics. NEC, not elsewhere classified.

**Table S2.** Reports distribution based on preferred terms (PT) classification.

| **Preferred terms** | **VEGFR-TKI + ICI** | | | **ICIs** | | **VEGFR-TKIs** | | |
| --- | --- | --- | --- | --- | --- | --- | --- | --- |
|  | **LP** | **AP** | **CN** | **P** | **N** | **A** | **C** | **L** |
| **Adrenal insufficiency, n** | 423 | 31 | 117 | 624 | 1363 | 41 | 72 | 37 |
| **Adrenocortical insufficiency acute, n** | 19 | 0 | 0 | 70 | 120 | 0 | 0 | 0 |
| **Immune-mediated adrenal insufficiency, n** | 49 | 0 | 0 | 142 | 155 | 0 | 0 | 0 |
| **Adrenal disorder, n** | 33 | 11 | 17 | 110 | 192 | 0 | 13 | 0 |
| **Metastases to adrenals, n** | 0 | 0 | 10 | 18 | 27 | 0 | 11 | 0 |
| **Hyperpituitarism, n** | 0 | 0 | 0 | 0 | 0 | 6 | 0 | 0 |
| **Hypopituitarism, n** | 54 | 9 | 10 | 197 | 665 | 14 | 18 | 59 |
| **Adrenocorticotropic hormone deficiency, n** | 39 | 5 | 7 | 301 | 471 | 5 | 8 | 42 |
| **Thyroid stimulating hormone deficiency, n** | 0 | 2 | 3 | 2 | 13 | 2 | 3 | 0 |
| **Growth hormone deficiency, n** | 0 | 0 | 0 | 0 | 0 | 0 | 0 | 1 |
| **Secondary hypogonadism, n** | 0 | 0 | 0 | 0 | 6 | 0 | 0 | 0 |
| **Hypothalamic pituitary adrenal axis suppression, n** | 0 | 0 | 0 | 0 | 5 | 0 | 0 | 0 |
| **Hypophysitis, n** | 38 | 6 | 19 | 308 | 805 | 8 | 29 | 40 |
| **Lymphocytic hypophysitis, n** | 3 | 0 | 3 | 27 | 120 | 0 | 5 | 5 |
| **Immune-mediated hypophysitis, n** | 4 | 2 | 2 | 0 | 160 | 3 | 0 | 5 |
| **Secondary adrenocortical insufficiency, n** | 15 | 6 | 0 | 152 | 472 | 11 | 5 | 17 |
| **Hypothalamo-pituitary disorder, n** | 11 | 11 | 4 | 154 | 306 | 14 | 0 | 11 |
| **Pituitary enlargement, n** | 0 | 1 | 0 | 6 | 32 | 0 | 0 | 0 |
| **Pituitary haemorrhage, n** | 0 | 0 | 0 | 0 | 5 | 0 | 0 | 2 |
| **Pituitary apoplexy, n** | 0 | 0 | 0 | 0 | 0 | 2 | 0 | 0 |
| **Empty sella syndrome, n** | 0 | 1 | 0 | 4 | 4 | 0 | 0 | 0 |
| **Growth disorder, n** | 0 | 0 | 0 | 0 | 0 | 0 | 0 | 1 |
| **Inappropriate antidiuretic hormone secretion, n** | 21 | 3 | 0 | 122 | 141 | 5 | 0 | 36 |
| **Diabetes insipidus, n** | 0 | 0 | 0 | 26 | 34 | 0 | 0 | 0 |
| **Polydipsia, n** | 0 | 0 | 0 | 0 | 9 | 0 | 0 | 0 |
| **Pituitary tumour, n** | 4 | 0 | 0 | 0 | 0 | 0 | 0 | 0 |
| **Pituitary tumour benign, n** | 0 | 0 | 0 | 20 | 6 | 0 | 0 | 0 |
| **Thyroid disorder, n** | 159 | 40 | 101 | 452 | 480 | 98 | 231 | 196 |
| **Euthyroid sick syndrome, n** | 0 | 0 | 0 | 0 | 4 | 0 | 0 | 0 |
| **Thyrotoxic crisis, n** | 17 | 3 | 0 | 37 | 45 | 4 | 0 | 20 |
| **Hyperthyroidism, n** | 257 | 35 | 95 | 739 | 946 | 72 | 203 | 311 |
| **Immune-mediated hyperthyroidism, n** | 27 | 6 | 3 | 112 | 62 | 6 | 3 | 27 |
| **Secondary hyperthyroidism, n** | 2 | 0 | 0 | 3 | 0 | 0 | 0 | 2 |
| **Basedow's disease, n** | 0 | 8 | 6 | 27 | 59 | 8 | 0 | 0 |
| **Graves' disease, n** | 0 | 4 | 5 | 7 | 17 | 4 | 5 | 0 |
| **Toxic nodular goitre, n** | 0 | 0 | 0 | 3 | 3 | 0 | 0 | 0 |
| **Exophthalmos, n** | 0 | 0 | 0 | 10 | 0 | 0 | 0 | 0 |
| **Hypothyroidism, n** | 1234 | 101 | 192 | 2300 | 1949 | 245 | 726 | 1544 |
| **Primary hypothyroidism, n** | 9 | 0 | 0 | 22 | 29 | 0 | 1 | 10 |
| **Immune-mediated hypothyroidism, n** | 197 | 15 | 6 | 531 | 240 | 15 | 10 | 197 |
| **Autoimmune hypothyroidism, n** | 2 | 0 | 0 | 56 | 39 | 0 | 0 | 0 |
| **Myxoedema coma, n** | 0 | 3 | 0 | 12 | 0 | 3 | 0 | 0 |
| **Myxoedema, n** | 0 | 0 | 0 | 10 | 0 | 0 | 0 | 0 |
| **Thyroid atrophy, n** | 0 | 0 | 0 | 0 | 1 | 0 | 0 | 0 |
| **Goitre, n** | 0 | 0 | 0 | 0 | 27 | 0 | 5 | 0 |
| **Thyroiditis, n** | 176 | 16 | 27 | 460 | 613 | 33 | 40 | 210 |
| **Immune-mediated thyroiditis, n** | 14 | 2 | 9 | 149 | 149 | 2 | 9 | 14 |
| **Silent thyroiditis, n** | 7 | 0 | 1 | 29 | 47 | 0 | 0 | 9 |
| **Thyroiditis subacute, n** | 12 | 0 | 4 | 17 | 22 | 0 | 6 | 12 |
| **Thyroiditis acute, n** | 7 | 0 | 0 | 8 | 6 | 2 | 0 | 7 |
| **Autoimmune thyroiditis, n** | 0 | 0 | 5 | 115 | 188 | 0 | 7 | 0 |
| **Atrophic thyroiditis, n** | 0 | 0 | 0 | 1 | 0 | 0 | 0 | 0 |
| **Thyroid injury, n** | 0 | 1 | 0 | 0 | 0 | 0 | 0 | 0 |
| **Autoimmune thyroid disorders, n** | 0 | 0 | 0 | 0 | 20 | 0 | 2 | 0 |
| **Infectious thyroiditis, n** | 0 | 0 | 0 | 0 | 0 | 0 | 2 | 0 |
| **Thyroid mass, n** | 0 | 0 | 0 | 0 | 0 | 0 | 0 | 6 |
| **Poorly differentiated thyroid carcinoma, n** | 3 | 0 | 0 | 3 | 0 | 0 | 0 | 3 |
| **Metastases to thyroid, n** | 0 | 0 | 0 | 0 | 4 | 0 | 0 | 0 |
| **Anaplastic thyroid cancer, n** | 0 | 0 | 0 | 3 | 3 | 0 | 0 | 4 |
| **Medullary thyroid cancer, n** | 0 | 0 | 0 | 0 | 0 | 0 | 5 | 0 |
| **Thyroid cancer metastatic, n** | 0 | 0 | 0 | 0 | 0 | 0 | 2 | 0 |
| **Thyroid cancer, n** | 0 | 0 | 0 | 6 | 0 | 0 | 1 | 13 |
| **Papillary thyroid cancer, n** | 0 | 0 | 0 | 0 | 0 | 0 | 0 | 5 |
| **Tetany, n** | 4 | 0 | 0 | 0 | 0 | 0 | 0 | 5 |
| **Parathyroid tumour malignant, n** | 0 | 0 | 0 | 4 | 0 | 0 | 0 | 0 |
| **Parathyroid tumour benign, n** | 0 | 0 | 0 | 7 | 0 | 0 | 0 | 0 |
| **Hypoparathyroidism, n** | 0 | 0 | 0 | 10 | 14 | 0 | 0 | 0 |
| **Primary hypoparathyroidism, n** | 0 | 0 | 0 | 2 | 0 | 0 | 0 | 0 |
| **Diabetes Mellitus, n** | 57 | 25 | 16 | 460 | 655 | 91 | 0 | 98 |
| **Type 1 diabetes mellitus, n** | 89 | 15 | 13 | 493 | 618 | 34 | 18 | 91 |
| **Fulminant type 1 diabetes mellitus, n** | 26 | 0 | 8 | 117 | 848 | 6 | 8 | 29 |
| **Latent autoimmune diabetes in adults, n** | 4 | 0 | 0 | 9 | 9 | 0 | 0 | 4 |
| **Type 2 diabetes mellitus, n** | 0 | 0 | 0 | 109 | 147 | 0 | 0 | 0 |
| **Insulin-requiring type 2 diabetes mellitus, n** | 0 | 0 | 0 | 3 | 0 | 0 | 0 | 0 |
| **Type 3 diabetes mellitus, n** | 0 | 0 | 0 | 0 | 11 | 0 | 0 | 0 |
| **Steroid diabetes, n** | 0 | 0 | 0 | 17 | 62 | 4 | 0 | 0 |
| **Diabetes mellitus inadequate control, n** | 10 | 0 | 0 | 67 | 0 | 0 | 0 | 18 |
| **Diabetes with hyperosmolarity, n** | 0 | 0 | 0 | 0 | 2 | 0 | 0 | 0 |
| **Hyperglycaemia, n** | 55 | 14 | 41 | 290 | 521 | 39 | 62 | 67 |
| **Glucose tolerance impaired, n** | 0 | 0 | 0 | 13 | 0 | 0 | 0 | 0 |
| **Impaired insulin secretion, n** | 0 | 0 | 0 | 0 | 6 | 0 | 0 | 0 |
| **Hypoglycaemia, n** | 27 | 19 | 30 | 115 | 200 | 31 | 80 | 52 |
| **Shock hypoglycaemic, n** | 3 | 0 | 0 | 3 | 0 | 0 | 0 | 3 |
| **Hypoglycaemic encephalopathy, n** | 2 | 0 | 0 | 0 | 0 | 0 | 0 | 2 |
| **Hypoglycaemic coma, n** | 0 | 0 | 0 | 1 | 7 | 0 | 0 | 0 |
| **Hypogonadism, n** | 0 | 0 | 0 | 9 | 14 | 0 | 0 | 0 |
| **Gonadotrophin deficiency, n** | 0 | 0 | 0 | 4 | 5 | 0 | 0 | 0 |
| **Amenorrhoea, n** | 0 | 0 | 0 | 4 | 3 | 0 | 0 | 0 |
| **Premature menopause, n** | 0 | 0 | 0 | 0 | 2 | 0 | 0 | 0 |
| **Neuroendocrine tumour, n** | 0 | 0 | 0 | 5 | 0 | 0 | 0 | 0 |
| **Neuroendocrine tumour of the lung metastatic, n** | 0 | 0 | 0 | 2 | 0 | 0 | 0 | 0 |
| **Hypercalcaemia of malignancy, n** | 0 | 0 | 5 | 0 | 15 | 0 | 6 | 0 |
| **Neuroendocrine carcinoma of the skin, n** | 0 | 0 | 0 | 0 | 7 | 0 | 0 | 0 |
| **Pancreatic neuroendocrine tumour, n** | 0 | 0 | 0 | 0 | 0 | 0 | 0 | 5 |
| **TOTAL, n** | **4,267** | | | **22,349** | | **5,624** | | |

N, number of events; A, axitinib; AP, axitinib + pembrolizumab; C, cabozantinib; CN, cabozantinib + nivolumab; L, lenvatinib; LP, lenvatinib + pembrolizumab; N, nivolumab; P, pembrolizumab.

**Table S3.** Adverse events with VEGFR-TKI + ICI therapy grouped according to High Level Group Term.

| **HLGT** | **N, n** | **%** |
| --- | --- | --- |
| Adrenal gland disorders | 5 | 10.41 |
| Hypothalamus and pituitary gland disorders | 12 | 25.00 |
| Thyroid gland disorders | 20 | 41.67 |
| Parathyroid gland disorders | 1 | 2.08 |
| Glucose metabolism disorders | 9 | 18.75 |
| Endocrine disorders of gonadal function | 0 | 0.00 |
| Neoplastic and ectopic endocrinopathies | 1 | 2.08 |

HLGT, High Level Group Term; N, number of events; %, percentage.

**Table S4.** Adverse events with VEGFR-TKI monotherapy grouped according to High Level Group Term.

| **HLGT** | **N, n** | **%** |
| --- | --- | --- |
| Adrenal gland disorders | 3 | 5.26 |
| Hypothalamus and pituitary gland disorders | 14 | 24.56 |
| Thyroid gland disorders | 27 | 47.37 |
| Parathyroid gland disorders | 1 | 1.75 |
| Glucose metabolism disorders | 10 | 17.54 |
| Endocrine disorders of gonadal function | 0 | 0.00 |
| Neoplastic and ectopic endocrinopathies | 2 | 3.51 |

**Table S5.** Adverse events with ICI monotherapy grouped according to High Level Group Term.

| **HLGT** | **N, n** | **%** |
| --- | --- | --- |
| Adrenal gland disorders | 5 | 6.25 |
| Hypothalamus and pituitary gland disorders | 17 | 21.25 |
| Thyroid gland disorders | 30 | 37.50 |
| Parathyroid gland disorders | 4 | 5.00 |
| Glucose metabolism disorders | 16 | 20.00 |
| Endocrine disorders of gonadal function | 4 | 5.00 |
| Neoplastic and ectopic endocrinopathies | 4 | 5.00 |

**Table S6.** Reporting odds ratio of endocrine adverse events requiring hospitalization for VEGFR-TKI + ICI vs. VEGFR-TKI monotherapy.

| **SOC: ENDOCRINE DISORDERS** | | | | | | | | | | | | |
| --- | --- | --- | --- | --- | --- | --- | --- | --- | --- | --- | --- | --- |
|  | **VEGFR-TKI + ICI vs VEGFR-TKI** | | | | | | | | | | | |
|  | **AP vs. A** | | | | **CN vs. C** | | | | **LP vs. L** | | | |
|  | **ROR** | **LCI** | **HCI** | **p** | **ROR** | **LCI** | **HCI** | **p** | **ROR** | **LCI** | **HCI** | **p** |
| **HLGT: ADRENAL GLAND DISORDERS** | | | | | | | | | | | | |
| **HLT: Adrenal cortical hypofunction** | | | | | | | | | | | | |
| **Adrenal insufficiency** | 2.06 | 1.24 | 5.49 | <0.05 | 8.15 | 5.71 | 11.74 | <0.01 | 22.99 | 14.39 | 36.73 | <0.01 |
| **HLT: Adrenal gland disorders nec** | | | | | | | | | | | | |
| **Adrenal disorder** | n.a. |  |  |  | 3.65 | 1.18 | 11.34 | <0.05 | n.a. |  |  |  |
| **HLT: Adrenal neoplasms** | | | | | | | | | | | | |
| **Metastases to adrenals** | n.a. |  |  |  | 6.40 | 1.87 | 21.9 | <0.01 | n.a. |  |  |  |
| **HLGT: HYPOTHLAMUS AND PITUITARY GLAND DISORDERS** | | | | | | | | | | | | |
| **HLT: Anterior pituitary hypofunction** | | | | | | | | | | | | |
| **Hypopituitarism** | n.s. |  |  |  | n.s. |  |  |  | 1.63 | 1.04 | 2.55 | <0.05 |
| **Adrenocorticotropic hormone deficiency** | n.s. |  |  |  | n.s. |  |  |  | n.s. |  |  |  |
| **Thyroid stimulating hormone deficiency** | n.a. |  |  |  | n.a. |  |  |  | n.a. |  |  |  |
| **HLT: Hypothalamus and pituitary gland disorders nec** | | | | | | | | | | | | |
| **Hypophysitis** | n.a. |  |  |  | 2.39 | 1.30 | 4.42 | <0.01 | 1.80 | 1.10 | 2.97 | <0.05 |
| **Hypothalamo-pituitary disorder** | n.s. |  |  |  | n.a. |  |  |  | n.s. |  |  |  |
| **HLGT: THYROID GLAND DISORDERS** | | | | | | | | | | | | |
| **HLT: Thyroid disorders nec** | | | | | | | | | | | | |
| **Thyroid disorder** | 1.58 | 1.09 | 2.28 | <0.05 | 2.50 | 1.98 | 3.16 | <0.01 | 3.26 | 2.64 | 4.02 | <0.01 |
| **HLT: Thyroid hyperfunction disorders** | | | | | | | | | | | | |
| **Hyperthyroidism** | 1.88 | 1.25 | 2.82 | <0.01 | 2.67 | 2.09 | 3.42 | <0.01 | 1.52 | 1.28 | 1.79 | <0.01 |
| **Immune-mediated hyperthyroidism** | 3.85 | 1.24 | 11.94 | <0.05 | 5.66 | 1.14 | 28.07 | <0.05 | 1.83 | 1.07 | 3.12 | <0.05 |
| **Basedow's disease** | 3.85 | 1.44 | 10.69 | <0.01 | n.a |  |  |  | n.a |  |  |  |
| **Graves' disease** | n.s. |  |  |  | 5.66 | 1.64 | 19.58 | <0.01 | n.a |  |  |  |
| **HLT: Thyroid hypofunction disorders** | | | | | | | | | | | | |
| **Hypothyroidism** | 1.61 | 1.27 | 2.03 | <0.01 | 1.51 | 1.29 | 1.78 | <0.01 | 1.49 | 1.38 | 1.61 | <0.01 |
| **Immune-mediated hypothyroidism** | 3.86 | 1.88 | 7.90 | <0.01 | 3.40 | 1.23 | 9.36 | <0.05 | 1.84 | 1.51 | 2.24 | <0.01 |
| **HLT: Acute and chronic thyroiditis** | | | | | | | | | | | | |
| **Thyroiditis** | 1.87 | 1.03 | 3.40 | <0.05 | 3.83 | 2.35 | 6.25 | <0.01 | 1.54 | 1.26 | 1.88 | <0.01 |
| **Immune-mediated thyroiditis** | n.s |  |  |  | 5.67 | 2.25 | 14.28 | <0.01 | n.s |  |  |  |
| **Thyroiditis subacute** | n.a |  |  |  | 3.78 | 1.07 | 13.38 | <0.05 | n.s |  |  |  |
| **Autoimmune thyroiditis** | n.a |  |  |  | 4.05 | 1.28 | 12.75 | <0.05 | n.a |  |  |  |
| **HLGT: GLUCOSE METABOLISM DISORDERS** | | | | | | | | | | | | |
| **HLT: Diabetes mellitus incl subtypes** | | | | | | | | | | | | |
| **Type 1 diabetes mellitus** | n.s. |  |  |  | 3.38 | 1.54 | 7.42 | <0.01 | 1.83 | 1.27 | 2.60 | <0.01 |
| **Fulminant type 1 diabetes mellitus** | n.a. |  |  |  | 3.65 | 1.06 | 12.63 | <0.05 | n.s. |  |  |  |
| **HLT: Hyperglycaemic conditions nec** | | | | | | | | | | | | |
| **Hyperglycaemia** | n.s. |  |  |  | 2.48 | 1.57 | 3.92 | <0.01 | n.s. |  |  |  |
| **HLT: Hypoglycaemic conditions nec** | | | | | | | | | | | | |
| **Hypoglycaemia** | n.s. |  |  |  | n.s. |  |  |  | n.s. |  |  |  |

SOC: System Organ Class. HLGT: High Level Group Term. HLT: High Level Term; AP: axitinib + pembrolizumab; CN: cabozantinib + nivolumab; N: nivolumab; LP: lenvatinib + pembrolizumab; P: pembrolizumab; ROR: reporting odds ratio; LCI: low confidence interval; HCI: high confidence interval; n.a.: not applicable (0 adverse events recorded for one of the two comparators); n.s.: not significant.

**Table S7.** Reporting odds ratio of endocrine adverse events requiring hospitalization for VEGFR-TKI + ICI vs. ICI monotherapy.

| **SOC: ENDOCRINE DISORDERS** | | | | | | | | |  | | | |
| --- | --- | --- | --- | --- | --- | --- | --- | --- | --- | --- | --- | --- |
|  | **VEGFR-TKI + ICI vs ICI** | | | | | | | | | | | |
|  | **AP vs. P** | | | | **CN vs. N** | | | | **LP vs. P** | | | |
|  | **ROR** | **LCI** | **HCI** | **p** | **ROR** | **LCI** | **HCI** | **p** | **ROR** | **LCI** | **HCI** | **p** |
| **HLGT: ADRENAL GLAND DISORDERS** | | | | | | | | | | | | |
| **HLT: Adrenal cortical hypofunction** | | | | | | | | | | | | |
| **Adrenal insufficiency** | 5.50 | 3.03 | 9.97 | <0.01 | 1.52 | 1.23 | 1.88 | <0.01 | 10.20 | 7.79 | 13.35 | <0.01 |
| **HLT: Adrenal gland disorders nec** | | | | | | | | | | | | |
| **Adrenal disorder** | 3.81 | 1.82 | 7.92 | <0.01 | n.s. |  |  |  | n.s. |  |  |  |
| **HLT: Adrenal neoplasms** | | | | | | | | | | | | |
| **Metastases to adrenals** | n.a. |  |  |  | 8.03 | 3.20 | 20.16 | <0.01 | n.a. |  |  |  |
| **HLGT: HYPOTHLAMUS AND PITUITARY GLAND DISORDERS** | | | | | | | | | | | | |
| **HLT: Anterior pituitary hypofunction** | | | | | | | | | | | | |
| **Hypopituitarism** | n.s. |  |  |  | 0.17 | 0.08 | 0.38 | <0.01 | n.s. |  |  |  |
| **Adrenocorticotropic hormone deficiency** | n.s. |  |  |  | 0.12 | 0.04 | 0.36 | <0.01 | 0.29 | 0.19 | 0.44 | <0.01 |
| **Thyroid stimulating hormone deficiency** | n.a. |  |  |  | n.a. |  |  |  | n.a. |  |  |  |
| **HLT: Hypothalamus and pituitary gland disorders nec** | | | | | | | | | | | | |
| **Hypophysitis** | n.a. |  |  |  | 0.43 | 0.27 | 0.70 | <0.01 | 0.53 | 0.36 | 0.78 | <0.01 |
| **Lymphocytic hypophysitis** | n.a. |  |  |  | n.s. |  |  |  | n.s. |  |  |  |
| **Immune-mediated hypophysitis** | n.a. |  |  |  | n.s. |  |  |  | n.a. |  |  |  |
| **Secondary adrenocortical insufficiency** | n.s. |  |  |  | n.a. |  |  |  | 0.26 | 0.14 | 0.49 | <0.01 |
| **Hypothalamo-pituitary disorder** | 3.30 | 1.52 | 7.30 | <0.01 | n.s. |  |  |  | 0.24 | 0.10 | 0.61 | <0.01 |
| **HLGT: THYROID GLAND DISORDERS** | | | | | | | | | | | | |
| **HLT: Thyroid disorders nec** | | | | | | | | | | | | |
| **Thyroid disorder** | n.s |  |  |  | 1.71 | 1.02 | 2.87 | <0.05 | n.s |  |  |  |
| **HLT: Thyroid hyperfunction disorders** | | | | | | | | | | | | |
| **Thyrotoxic crisis** | n.s. |  |  |  | n.a. |  |  |  | n.s. |  |  |  |
| **Hyperthyroidism** | n.s |  |  |  | 2.18 | 1.54 | 3.08 | <0.01 | 1.52 | 1.22 | 1.89 | <0.01 |
| **Basedow's disease** | n.a. |  |  |  | n.s. |  |  |  | n.a. |  |  |  |
| **Graves' disease** | n.a. |  |  |  | n.a. |  |  |  | n.a. |  |  |  |
| **HLT: Thyroid hypofunction disorders** | | | | | | | | | | | | |
| **Hypothyroidism** | n.s |  |  |  | n.s |  |  |  | 1.47 | 1.33 | 1.63 | <0.01 |
| **Immune-mediated hypothyroidism** | 3.00 | 1.19 | 7.58 | <0.05 | n.s |  |  |  | 1.86 | 1.44 | 2.41 | <0.01 |
| **Autoimmune hypothyroidism** | n.a. |  |  |  | n.a. |  |  |  | n.a. |  |  |  |
| **Myxoedema coma** | 7.24 | 2.04 | 25.70 | <0.01 | n.a. |  |  |  | n.a. |  |  |  |
| **HLT: Acute and chronic thyroiditis** | | | | | | | | | | | | |
| **Thyroiditis** | n.s |  |  |  | 2.52 | 1.66 | 5.43 | <0.05 | 1.65 | 1.27 | 2.14 | <0.01 |
| **Immune-mediated thyroiditis** | n.s |  |  |  | 3.66 | 1.37 | 9.75 | <0.01 | n.s |  |  |  |
| **Thyroiditis subacute** | n.a. |  |  |  | n.s. |  |  |  | n.s. |  |  |  |
| **Thyroiditis acute** | n.a. |  |  |  | n.s. |  |  |  | n.s. |  |  |  |
| **Autoimmune thyroiditis** | n.a. |  |  |  | n.a. |  |  |  | n.a. |  |  |  |
| **HLGT: GLUCOSE METABOLISM DISORDERS** | | | | | | | | | | | | |
| **HLT: Diabetes mellitus incl subtypes** | | | | | | | | | | | | |
| **Diabetes mellitus** | n.s. |  |  |  | 0.29 | 0.14 | 0.62 | <0.01 | 0.41 | 0.28 | 0.59 | <0.01 |
| **Type 1 diabetes mellitus** | n.s. |  |  |  | 0.44 | 0.24 | 0.78 | <0.01 | 0.63 | 0.47 | 0.84 | <0.01 |
| **Fulminant type 1 diabetes mellitus** | n.a. |  |  |  | 0.11 | 0.05 | 0.27 | <0.01 | n.s. |  |  |  |
| **HLT: Hypoglycaemic conditions nec** | | | | | | | | | | | | |
| **Hypoglycaemia** | 4.26 | 2.37 | 7.64 | <0.01 | 1.91 | 1.21 | 3.02 | <0.01 | n.s. |  |  |  |

n.a.: not applicable (0 adverse events recorded for one of the two comparators); n.s.: not significant.
